# Investigation on the fault monitoring of high-voltage circuit breaker using improved deep learning

**DOI:** 10.1371/journal.pone.0295278

**Published:** 2023-12-01

**Authors:** Hao Chen, Chenlei Han, Yucheng Zhang, Zhaoxing Ma, Haihua Zhang, Zhengxi Yuan

**Affiliations:** 1 State Grid Jiangsu Electric Power Co., Ltd. Nanjing Power Supply Branch, Nanjing, China; 2 School of Information and Control Engineering, Qingdao University of Technology, Qingdao, China; 3 School of Electrical Engineering, Southeast University, Nanjing, China; CINVESTAV IPN: Centro de Investigacion y de Estudios Avanzados del Instituto Politecnico Nacional, MEXICO

## Abstract

Mechanical faults are the main causes of abnormal opening, refusal operation, or malfunction of high-voltage circuit breakers. Accurately assessing the operational condition of high-voltage circuit breakers and delivering fault evaluations is essential for the power grid’s safety and reliability. This article develops a circuit breaker fault monitoring device, which diagnoses the mechanical faults of the circuit breaker by monitoring the vibration information data. At the same time, the article adopts an improved deep learning method to train vibration information of high-voltage circuit breakers, and based on this, a systematic research method is employed to identify circuit breaker faults. Firstly, vibration information data of high-voltage circuit breakers is obtained through monitoring devices, this vibration data is then trained using deep learning methods to extract features corresponding to various fault types. Secondly, using the extracted features, circuit breaker faults are classified and recognized with a systematic analysis of the progression traits across various fault categories. Finally, the circuit breaker’s fault type is ascertained by comparing the test set’s characteristics with those of the training set, using the vibration data. The experimental results show that for the same type of circuit breaker, the accuracy of this method is over 95%, providing a more efficient, intuitive, and practical method for online diagnosis and fault warning of high-voltage circuit breakers.

## 1. Introduction

While the technology of long-distance and large-capacity power transmission is undergoing rapid development, so did that of high-voltage transmission, evidenced by the introduction of ultra-high voltage electrical devices. Many high-voltage electrical devices have also been widely applied, among which high-voltage circuit breakers, as the most widely used switchgear devices in high-voltage power grid [1, 2], remain crucial for the safe and reliable operation of power system. However, their widespread use has brought challenges, such as the demand for scheduled power outage maintenance of high-voltage circuit breakers in use, which impedes power supply. In addition, accidents caused by its own operational characteristics account for over 60% of power outages both in terms of frequency and duration, which causes more devices life loss and power grid outage loss. Therefore, research on fault diagnosis of high-voltage circuit breakers has become increasingly important and exhibits significant application value. Many academics in this regard have done a great deal of research on fault detecting methods for high-voltage circuit breakers [3, 4], which have become a research hotspot in the academic and engineering fields. This paper will employ deep learning theory to perceive the operational states of high-voltage circuit breakers using vibration voiceprint data, .

Typical Mechanical failures of high-voltage circuit breakers include loose components, jammed iron cores, operating mechanism faults and so on. The commonly used method is to analyze the amplitude changes caused by above faults to characterize the operating states and achieve fault monitoring of circuit breakers. However, due to the complicated internal structure of high-voltage circuit breakers, vibration voiceprint data contains multitudinous information, which brings great difficulties to fault diagnosis and generate many unsolved problems in practical application. Therefore, devicing a high-performance detection model and effective identification method of the obtained voiceprint data pivotal for mechanical fault diagnosis of circuit breakers.

The detection methods for high-voltage circuit breakers can be roughly divided into offline and online detection. Among which, detecting the operating states of the circuit breaker through vibration signal is a non-invasive online detection method that does not affect the online operation of the circuit breaker, or damage the equipment. It is easier to implement and has received widespread attention from domestic and abroad [5–7]. The main methods for feature extraction of high-voltage circuit breaker vibration information data include envelope spectrum analysis [8], empirical mode decomposition [9], information entropy [10], wavelet packet analysis [11], phase space reconstruction [12], deep learning method [13], artificial intelligence [14, 15], etc. Compared with other methods, deep learning method can maintain high resolution throughout the entire frequency range [16, 17], extract vibration signal features through a large amount of training and learning of data from various fault types and characterize information from different time periods and frequency bands with high accuracy. For mechanical fault diagnosis of circuit breakers, a signal process system using short-term energy algorithms and timing extraction algorithms has been developed [18]. By setting statistical empirical thresholds, the identification of mechanical failures in circuit breakers was achieved. Based on empirical mode decomposition and information entropy theory, a fault diagnosis algorithm for high-voltage circuit breaker vibration signal was proposed [19], which is used to analyze the circuit breaker operating state, such as stuck or loose firmware, etc., and had achieved certain results.

For mechanical failure monitoring of high-voltage circuit breakers, obtaining accurate information on the opening and closing of the circuit breaker is very crucial for determining the fault type. In practical applications, when the device functions at high voltage, there is a significant deviation of the main circuit vibration data at the time of energization [20, 21], which leads to low accuracy of the stroke displacement curve and high misjudgment rate. When the circuit breaker undergoes mechanical vibration, the collected signals are susceptible to external electromagnetic environments [22, 23], which leads to a high error rate of fault identification and renders the collected signals unpractical. Based on the status quo, existing monitoring methods often have problems such as difficulty in collecting raw data, low identification accuracy and poor utilization of collected data, which affects the normal operation of devices. Therefore, a simpler and more practical fault monitoring method for circuit breaker becomes ever more pressing.

To overcome the shortcomings of the above methods and achieve accurate fault monitoring of high-voltage circuit breakers under small sample conditions, a fault monitoring method for high-voltage circuit breakers based on unsupervised deep learning theory is proposed in this paper. The content of the paper includes: vibration data collection, fault feature extraction as well as fault diagnosis. The second section analyzes the types of circuit breaker fault and monitoring model. The third section presents a model of deep learning algorithm, which trains data, extracts deep features, applies deep features to identify fault types and completes model training. In the fourth section, deep learning theory is used to monitor the application process of high-voltage circuit breakers while carrying out relevant experiments as well as obtaining a large amount of operating vibration data which is used to verify the validity and effectiveness of the theoretical analysis method proposed. The fifth part summarizes the full text.

## 2. Circuit breaker fault types and vibration monitoring system

### 2.1. Analysis of fault types and characteristics

The mechanical faults of high-voltage circuit breakers mainly include component looseness, iron core jamming, spring fatigue, and other types. The vibration signal frequency is relatively high. To simplify the acquisition of vibration information data, this article combines the structure of the circuit breaker and the opening and closing vibration situation, and selects the LC01 series LC0158T sensor to collect vibration data.

Mechanical faults are the core of circuit breaker failures and pose the most challenges for fault monitoring in this field. According to the type of fault, circuit breakers also exhibit typical fault characteristics, expressed as follows:

1. Dispersion of faults: Due to the complex coupling of its components and the randomness of faults, circuit breakers may experience faults in uncertain components, exhibiting both dispersion and uncertainty of faults.
2. Fault integrity: Due to the interest dependence of various components, circuit breakers may exhibit overall fault integrity due to different components. If certain components fail, it may cause overall/system-wide faults.

Due to the types and characteristics of circuit breaker faults, monitoring the vibration information displayed during faults can distinctly indicate fault types. Therefore, obtaining vibration information for fault type diagnosis is feasible and effective.

### 2.2. Monitoring system design

**Fig 1:**
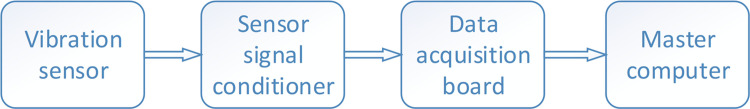
Information collection system for vibration data of circuit breaker.

The monitoring system includes vibration sensors, signal adaptors, data acquisition cards, and upper computers. The vibration sensor is arranged at the detection position of the high-voltage circuit breaker and connected to the signal adapter through a high-frequency shielded cable. The signal adapter is connected to the data acquisition card through a coaxial cable, which transmits the digital signal to the upper computer for storage. Then, an improved deep learning algorithm is used to analyze the obtained vibration information data and draw a diagnostic conclusion. The actual usage requirements of circuit breakers determine that there are differences in the operational requirements of circuit breakers among different functional sections. Therefore, the accuracy and focus of fault diagnosis also differ. The monitoring system framework for high-voltage circuit breaker vibration is shown in Fig 1.

**Fig 2:**
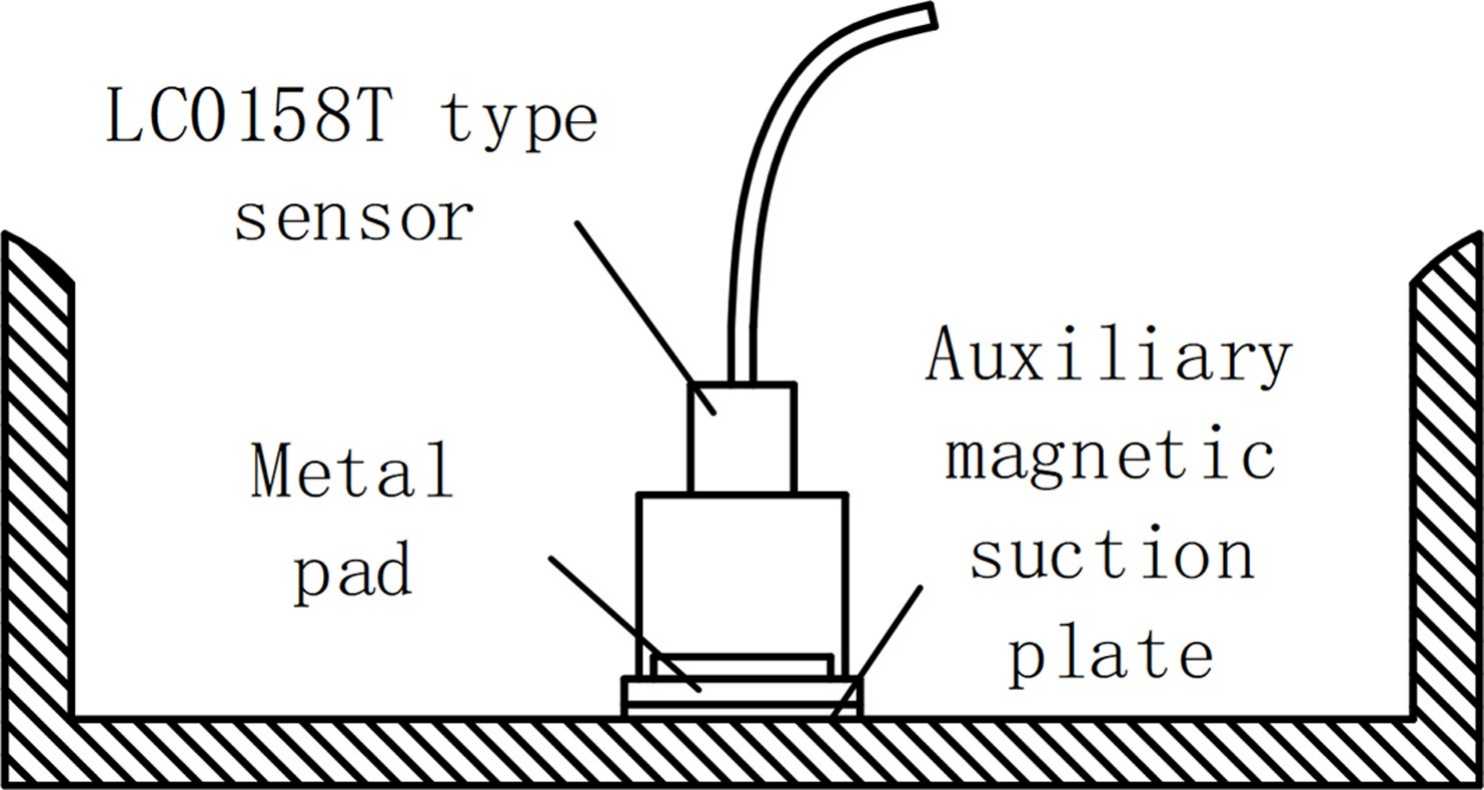
LC0158T model sensor.

In this study, the developed vibration information monitoring equipment selected acceleration sensors to capture the vibration signal of the circuit breaker. Considering sensitivity and sampling frequency, three LC01 series LC0158T sensors were used to collect vibration signals, as shown in Fig 2. The vibration sensor of this model has a range of 166g, a sensitivity of -25mV/g, and a frequency range of 0.007-50kHz.

Considering the peak value of the opening and closing coil current and the required measurement accuracy, two LA25-NP current transformers were selected to measure the opening and closing coil currents respectively. The current transformer has an accuracy of ± 0.5% and a range of (-5-+5A).

**Fig 3:**
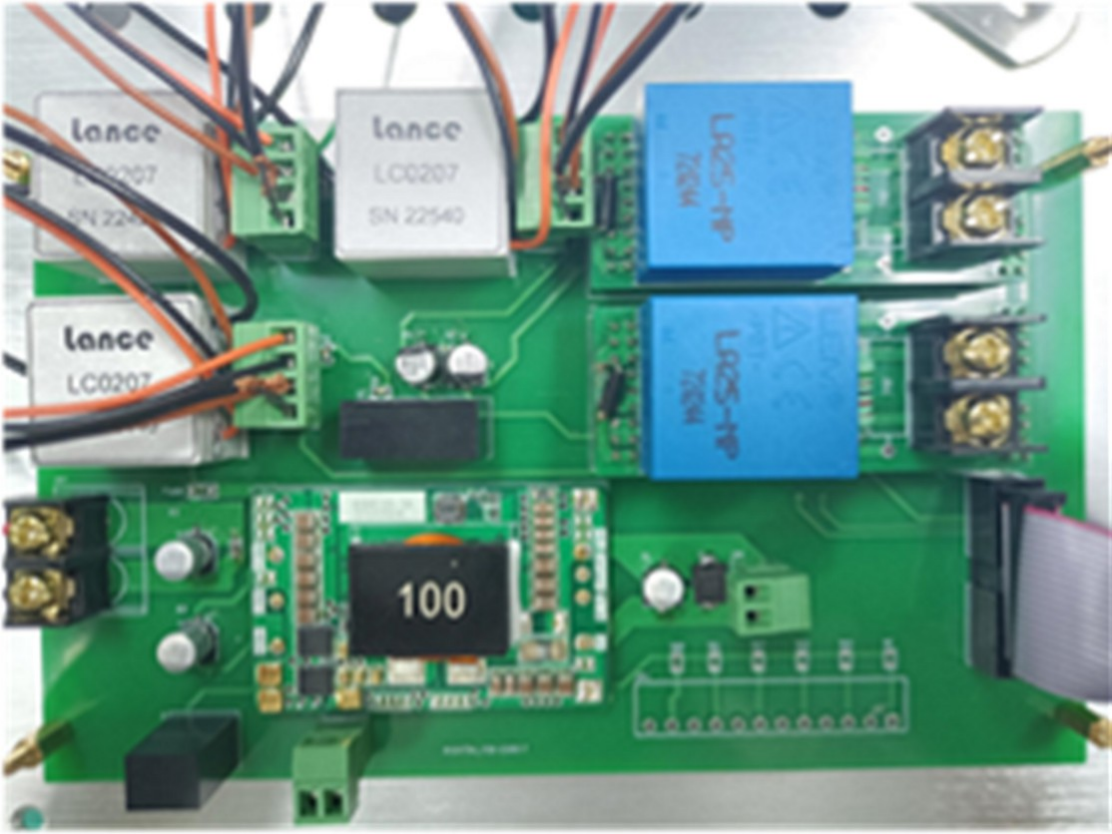
Physical image of PCB board.

The signal conditioning circuit board is designed using Altium Designer, and the circuit PCB board is shown in Fig 3.

The signal is inputted into the MP4221 data acquisition card through a signal conditioning circuit. The acquisition card has 8 analog inputs, supports 12 bit 2MHz AD, and is equipped with 8M FIFO memory. The signal acquisition buffer can reach 200ms. The data collection system uses software triggering and a storage fixed length collection method to collect data. The acquisiton card interfaces with the industrial computer via the USB2.0 bus.

Considering the performance of the industrial control computer and the convenience of subsequent device integration, the OTR-5612 industrial control computer was selected for development, and its configuration information is shown in Table 1.

**Table 1:** Industrial computer configuration information.

Item	Parameter	Item	Parameter
CPU	Intel Core i5-4200U	power supply	DC IN 12V
BIOS	AMIBIOS, ACIPSUPPORTED	Hard disk	Support for MSTA3 SSD
Graphics card	Integrated HD graphics card, supporting VGA+HDML simultaneous/asynchronous display	USB	4 USB3.0 3 USB2.0
Third level cache	3MB, CPU INTEGRATED	Network card	1 Gigabit network card
Size	234mm*150mm*52mm	Consumption	20-25W
Serial port	2 COM serial ports: RS232	Weight	About 1.5kg

**Fig 4:**
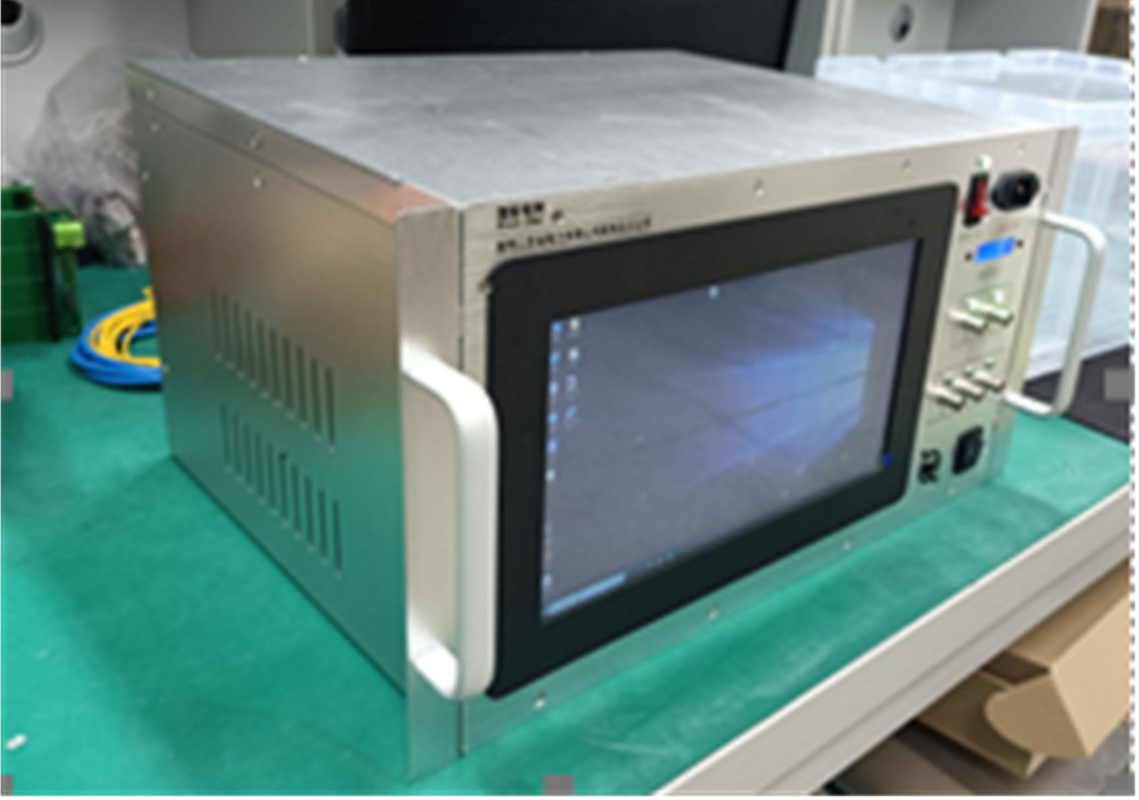
Vibration information collection device for mobile circuit breaker.

To combine various functional modules, panel components well installed such as 11.6-inch touchable industrial display screen, power switch, ship type switch, power display module, indicator light, coaxial cable connector, network port, and USB interface. After connecting the components on the panel and lining, fixing the liner and panel inside the chassis and installing handles as well as other chassis accessories. A mobile circuit breaker vibration information data acquisition device has been developed, as shown in Fig 4.

## 3. Improved deep learning algorithm

### 3.1. Data preprocessing and feature reconstruction

The accuracy of circuit breaker fault diagnosis largely depends on the feature extraction quality of deep belief network. This section will provide data preprocessing and unsupervised feature reconstruction methods to achieve feature extraction of circuit breaker vibration voiceprint.

The deep belief network is composed of multiple stacked restricted Boltzmann machines. One restricted Boltzmann machine consists of *n* visible layer units and *m* hidden layer units, its energy function is represented as follows:

$$E\left(k,g\left(\delta \right)\right)=\sum_{i=1}^{m}a_{i}k_{i}+\sum_{j=1}^{n}b_{j}g_{j}+\sum_{i=1}^{m}\sum_{j=1}^{n}k_{i}v_{ij}g_{j}$$ (1)

Where {*k*_*i*_ ∈ R|0 ≤ *k*_*i*_ ≤ 1}is state of the *i*th visible layer neuron. {*g*_*j*_ ∈ R|0 ≤ *g*_*j*_ ≤ 1} is state of the *j-*th hidden layer neuron. *a*_*i*_ is bias of visible layer unit *k*_*i*_. *b*_*j*_ is bias of the hidden layer unit *g*_*j*_. *v*_*ij*_ is connection weight between *k*_*i*_ and *g*_*j*_. *δ* is relevant to the network parameter *a*_*i*_, *b*_*j*_ and *v*_*ij*_.

**Fig 5:**
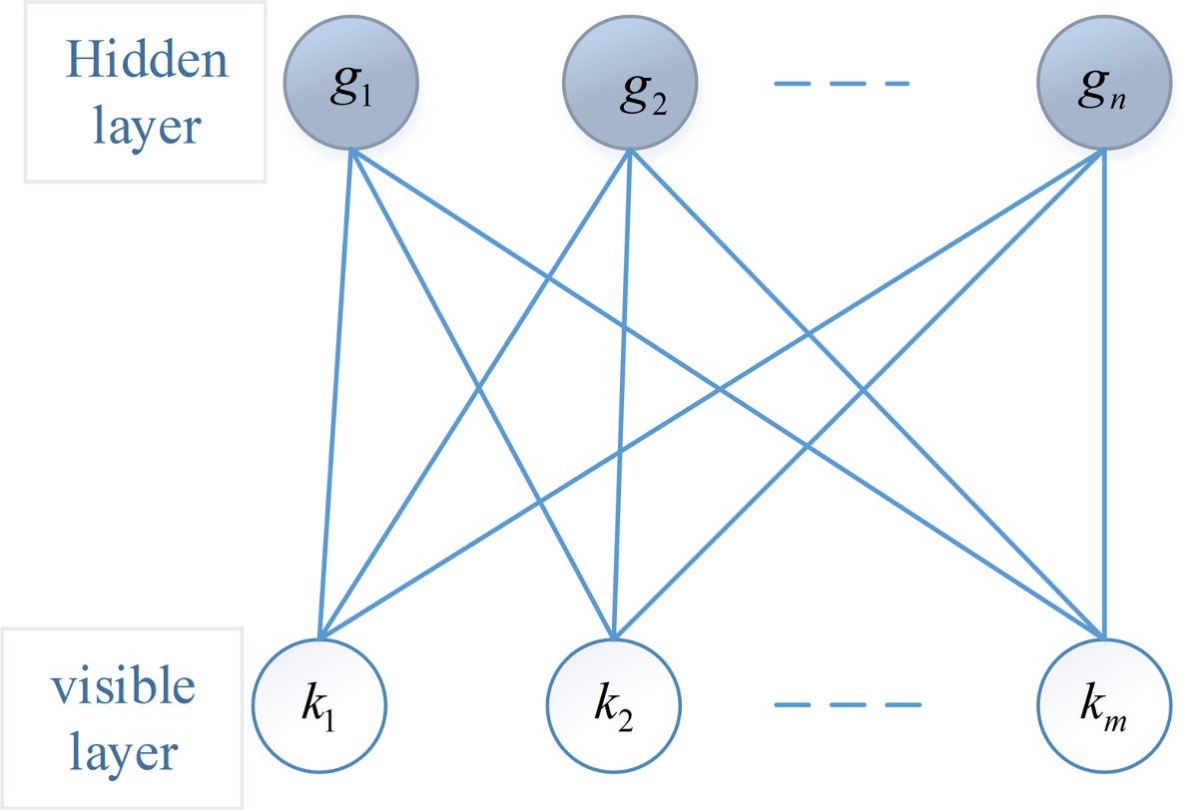
Structure of restricted Boltzmann machine model.

The basic structure of the restricted Boltzmann machine model is shown in Fig 5.

The joint probability density distribution function between the visible layer and the hidden layer is shown as follows:

$$f\left(k,g\right)=\frac{1}{C}e^{-E\left(k,g\left(\delta \right)\right)}$$ (2)

Where *C* is normalization factor.

The independent distribution function of the visual layer vector *k* is represented as follows:

$$f\left(k\right)=\sum_{g}f\left(k,g\right)=\frac{1}{C}\sum_{g}e^{-E\left(k,g\left(\delta \right)\right)}$$ (3)

The activation probabilities function of neurons in the hidden and visible layers are represented as follows:

$$f\left(g_{i}\;=\;1|k\right)\;=\;sig\mathrm{mo}id\left(a_{i}\;+\;\sum_{j=1}^{m}v_{ij}k_{j}\right)$$ (4a)

$$f\left(k_{j}=1|g\right)=sig\mathrm{mo}id\left(b_{j}+\sum_{i=1}^{n}v_{ij}g_{i}\right)$$ (4b)

When the value of *f*(*k*) reaches maximum, the probability distribution under the restricted Boltzmann machine structure is relatively consistent with the input data. The pretraining optimization objective function could be described as follows:

$$\max F=\prod_{k}f\left(k\right)$$ (5)

To improve computational efficiency, the gradient descent method is used to search for the optimal parameters of the network *δ*, of which the update iteration formulas are represented as follows:

$$a_{i}=a_{i}\;-\;\tau \frac{\partial E\left(k,g\left(\delta \right)\right)}{\partial a_{i}}$$ (6a)

$$b_{j}=b_{j}\;-\;\tau \frac{\partial E\left(k,g\left(\delta \right)\right)}{\partial b_{j}}$$ (6b)

$$v_{ij}=v_{ij}\;-\;\tau \frac{\partial E\left(k,g\left(\delta \right)\right)}{\partial v_{ij}}$$ (6c)

Where *τ* is learning rate.

**Fig 6:**
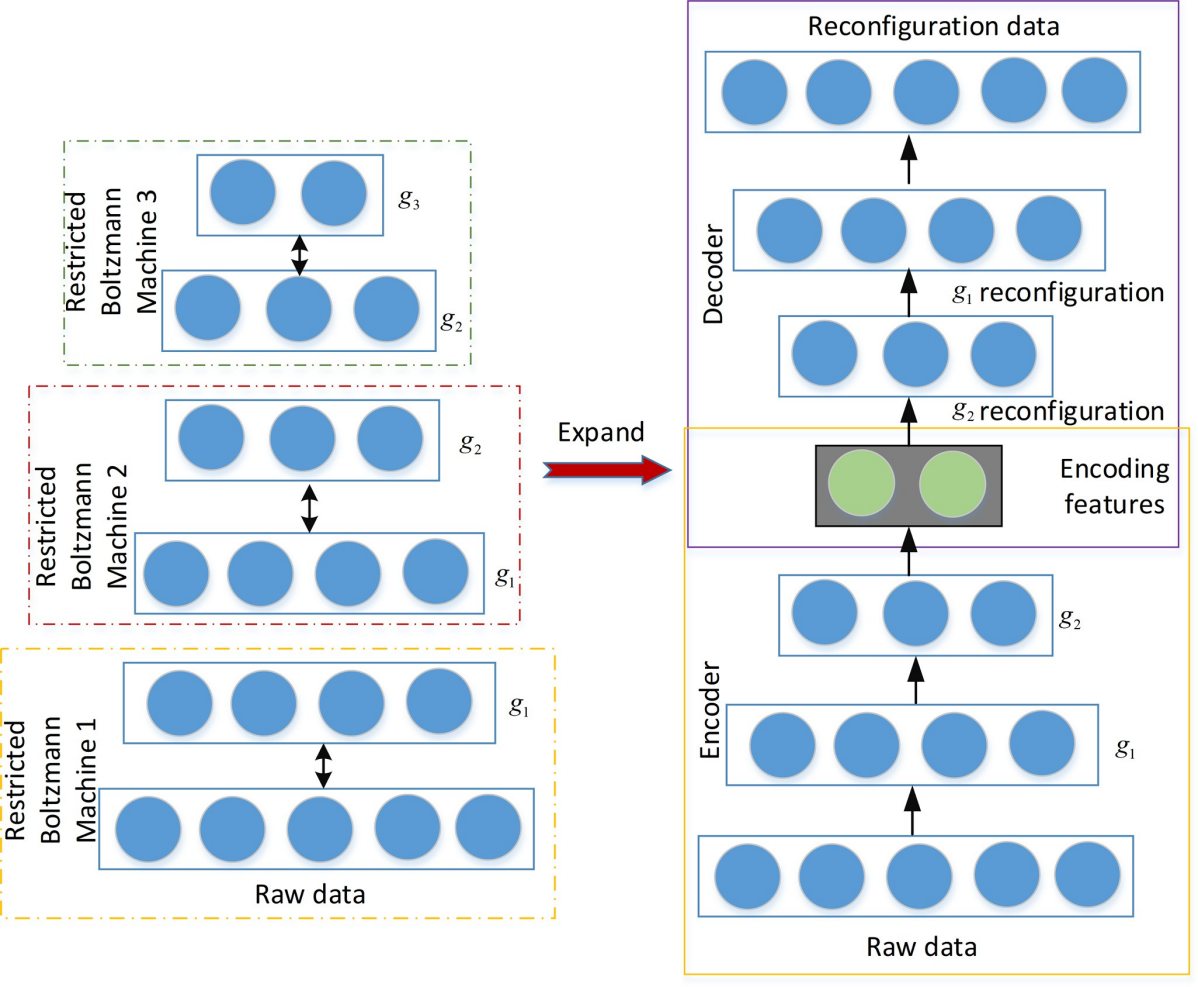
Reconstruction diagram of calculation method.

After the unsupervised learning process of the restricted Boltzmann machine, the output feature of original data can be observed. The output at the highest level does not go through the classifier, the hidden layer reconfiguration data is directly expanded layer by layer, and a self encoder is established. Different from the traditional deep belief network, the method given in this paper is unsupervised learning, meaning that the encoding and decoding process has consistent input and output dimensions and does not involve tagged data. The reconfiguration process is shown in Fig 6.

The forward learning process of a restricted Boltzmann machine is an encoding process, and its encoding form is represented by the following equation, which could be described as follow:

$$g=\frac{1}{1+\exp\left(-vs-a\right)}$$ (7)

Where *s* is input. *a* is bias parameter, and *v* is connection weight.

The reconstruction of hidden layer units is a decoding process and the corresponding decoding function is represented as follows:

$$s=\frac{1}{1+\exp\left(-v^{T}s-e\right)}$$ (8)

Where *e* is bias parameter.

The encoding and decoding process of the belief network generates a certain difference between the reconstructed and the original signal, of which the amplitude is inversely proportional to the feature extraction quality. To address this problem, a cost function measuring the difference between reconstructed and original data is provided in this paper, which could be described as follows:

$$B\left(s\right)=\frac{1}{T}{\sum_{t}^{T}\left|S_{t}^{l}\;-\;S_{t}\right|}^{2}$$ (9)

Where *T* is the number of samples. $S_{t}^{l}$ is the encoding output of the *t*-th sample after being subjected to the *l*-th layer stacking restricted Boltzmann machine. *S*_*t*_ is the raw data of the *t-*th sample.

**Fig 7:**
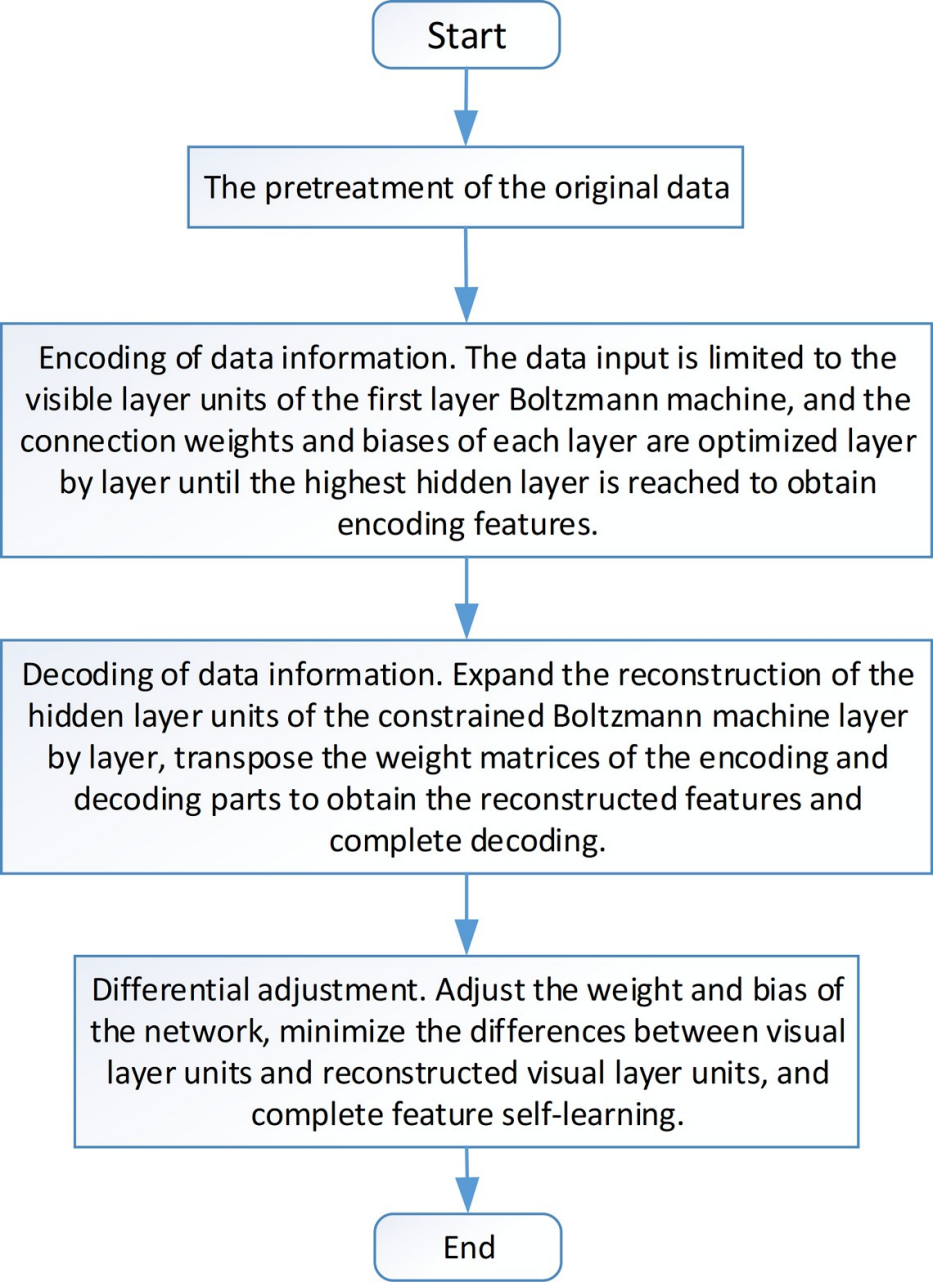
Feature reconstruction process of voiceprint data.

To improve the feature extraction quality, this paper uses BP algorithm for parameter optimization [24], applies the gradient descent method to obtain the partial derivative of *B*(*s*) with respect to parameters *a*, *e*, and *v*, then adjusts parameters *a*, *e*, and *v* accordingly to reduce the value of *B*(*s*) and improves the model’s ability to extract original features. Based on the previous discussion, the feature reconstruction steps provided in the paper are illustrated in Fig 7.

### 3.2. Data classification and retraining

After completing feature coding, classification layers and labels at the top level are needed to diagnose the circuit breaker faults. This section will provide learning training method for the classification layer. As was discussed before, the initial weights and biases of the restricted Boltzmann machine at the bottom layer are obtained from unsupervised training, and the softmax model is used as the classifier at the top layer.

Assuming the number of restricted Boltzmann machines in the deep belief network is d, and the deep belief network comprises d restricted Boltzmann machines [25, 26], the output vector of the last layer of restricted Boltzmann machines could be described as follows:

$$Q_{d}\left(s\right)=\frac{1}{1+\exp\left(v_{d}Q_{d-1}\left(s\right)+a_{d}\right)}$$ (10)

Where *s* is initial sample, and other parameters are shown in Eqs (8) and (1).

The *i*-th sample, after learning by a forward *d*-layer constrained Boltzmann machine, belongs to category *z*_*i*_, and the probability of *z*_*i*_ is expressed as:

$$P\left(z_{i}=r\right)=\frac{e^{k_{d}^{r}Q_{d}\left(s_{d}\right)}}{\sum_{r=1}^{R}e^{k_{d}^{r}Q_{d}\left(s_{d}\right)}}$$ (11)

Where *z*_*i*_∈{1,2,…,R}, $k_{d}^{r}$ is parameter coefficient which selects the category with the highest probability as the classifier output. Other parameters are shown in Eqs (8) and (10).

The cost function of the *l*-th layer of the belief network is represented as follows:

$$B\left(\varphi_{l}\right)=-\frac{1}{n}\left[\sum_{j=1}^{n}\sum_{r=1}^{R}1\left(z_{i}=r\right)\text{lg}\;p\left(z_{i}=r\right)\right]$$ (12)

Where 1(*z*_*i*_ = *r*) is logical indicator function. As *z*_*i*_ = *r*, the value is 1, otherwise the value is 0. *φ*_*l*_ = {*v*_*l*_, *a*_*l*_, *e*_*l*_, *k*_*l*_}.

In order to minimize the cost function value, as depicted in Eq (12), the error between the network output and the real numerical value is propagated backwards from the top layer to the bottom layer, which leads to repeated parameter adjustments until the error meets the requirement and completes the process.

**Fig 8:**
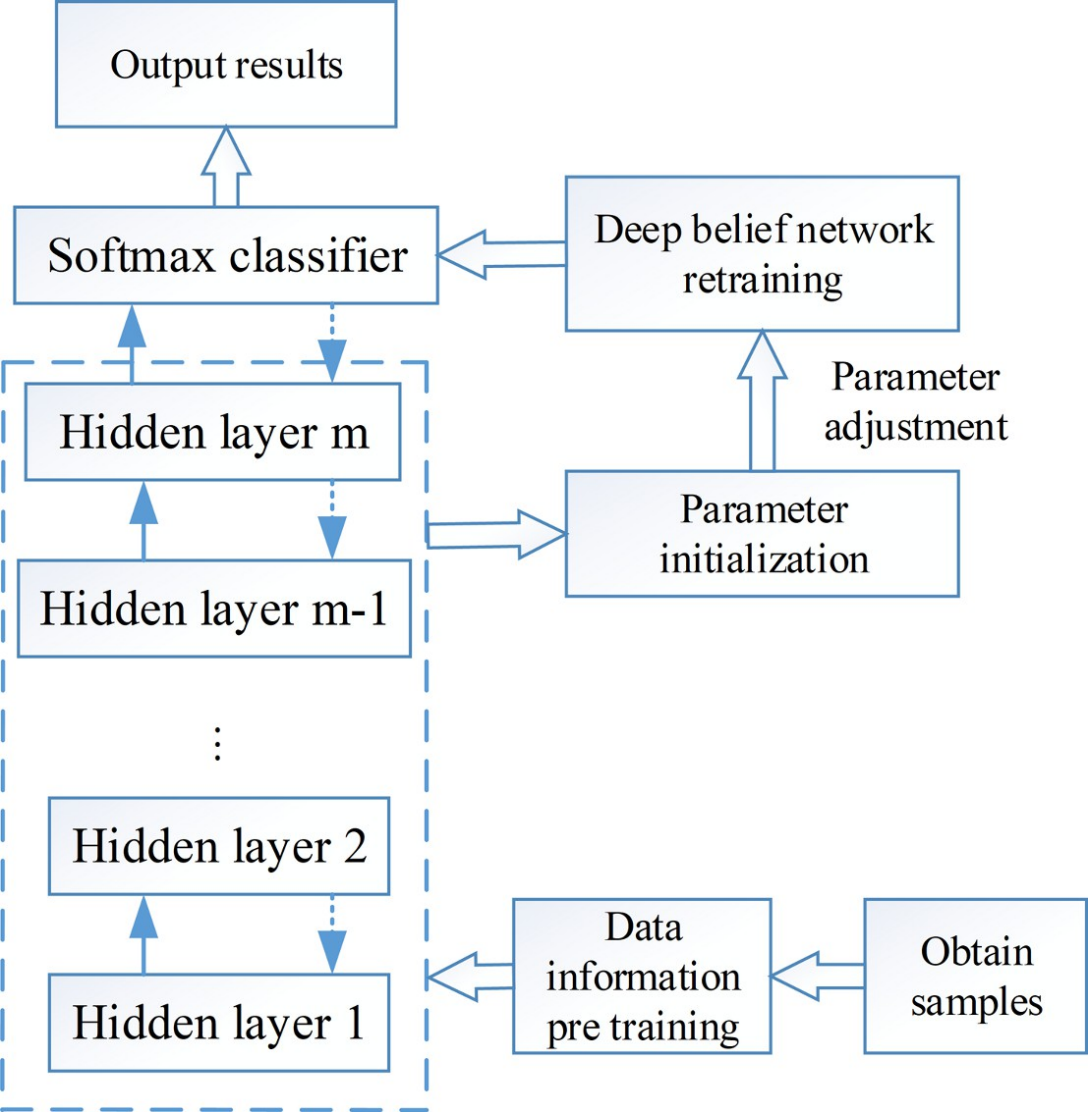
The training process of deep belief network model.

The feature extraction algorithm proposed in this paper is completed by adding tag data to the retraining process based on the bottom restricted Boltzmann machine and unsupervised learning. The training process is shown in Fig 8.

### 3.3. Process of vibration feature extraction and fault diagnosis

Based on the above analysis, this article proposes a detailed process for detecting circuit breaker faults using based on the characteristics of vibration voiceprint, as described below.

Step 1. Obtain the vibration voiceprint data of the circuit breaker, then define and encode the vibration voiceprint data.
Step 2. Build an analysis model and data package. Collect and preprocess vibration voiceprint data sample and delineate different datasets of vibration voiceprint based on fault types.
Step 3. Build a training model. Construct a restricted Boltzmann machine model to determine the number of input layer nodes and connection weights.
Step 4. Extracte the vibration voiceprint data feature. Train the detected data training package layer by layer so that the sample data can represent the true state of the circuit breaker and extract data features. Meanwhile, establish an autoencoder to reconstruct input features.
Step 5. Utilize labeled training sets to retrain the restricted Boltzmann machine model and the softmax classification layer, adjusts the corresponding weights and biases.
Step 6. Input the test data of circuit breaker vibration voiceprint into the trained deep belief network for fault diagnosis and provide the diagnosis results.

## 4. Example verification and analysis

### 4.1. Building an experimental platform

This section uses the vibration information data of YFGZ35 (EP) -40.5 circuit breaker as training data. Through artificial fault simulation tests, training datasets of different fault types are obtained with a sampling interval of 1ms. The technical parameters of the vibration sensor are shown in Table 2.

**Table 2:** Technical parameters of vibration sensor.

Parameters	Value
Charge sensitivity	0.03m•s^-2^
Maximum impact acceleration	50000 m•s^-2^
Frequency response	0∼15000Hz
Resonant response	45kHz

**Fig 9:**
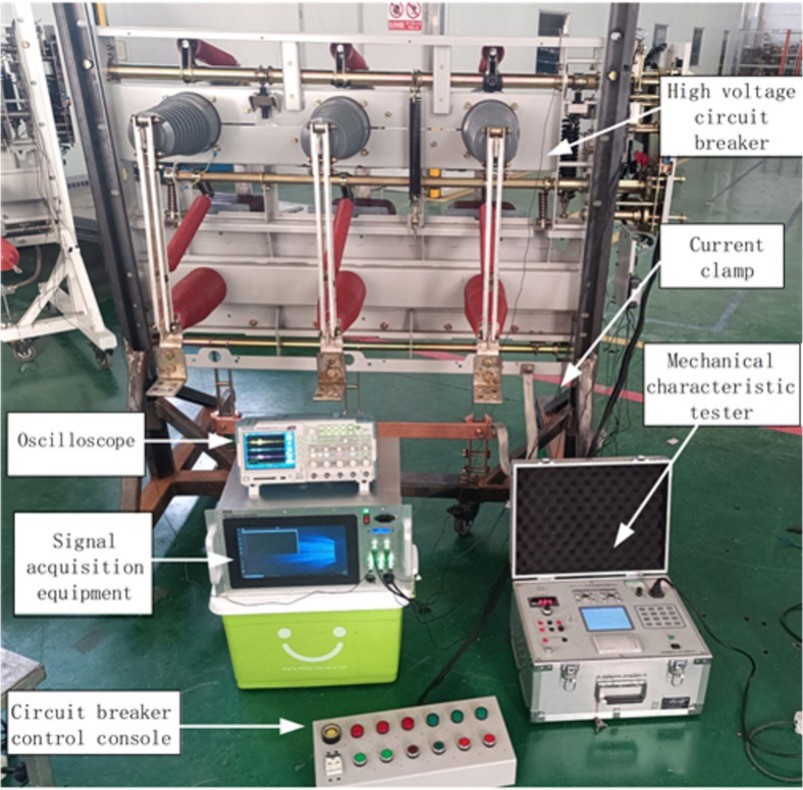
Data acquisition test system.

This article selects YFGZ35 (EP) -40.5 circuit breaker as the tested circuit breaker for laboratory simulation testing. The experimental system, as shown in Fig 9, includes circuit breakers, vibration sensors, current transformers, mobile devices, circuit breaker consoles, and mechanical characteristic testers for comparison.

Through the circuit breaker console, initiate manual operations for opening and closing. When the mobile device’s software system detects a sampling value surpassing the threshold, it activates an interrupt subroutine. This procedure then consistently collects a signal for 150ms, stores it in a data file, and proceeds to further processing and analysis.

**Fig 10:**
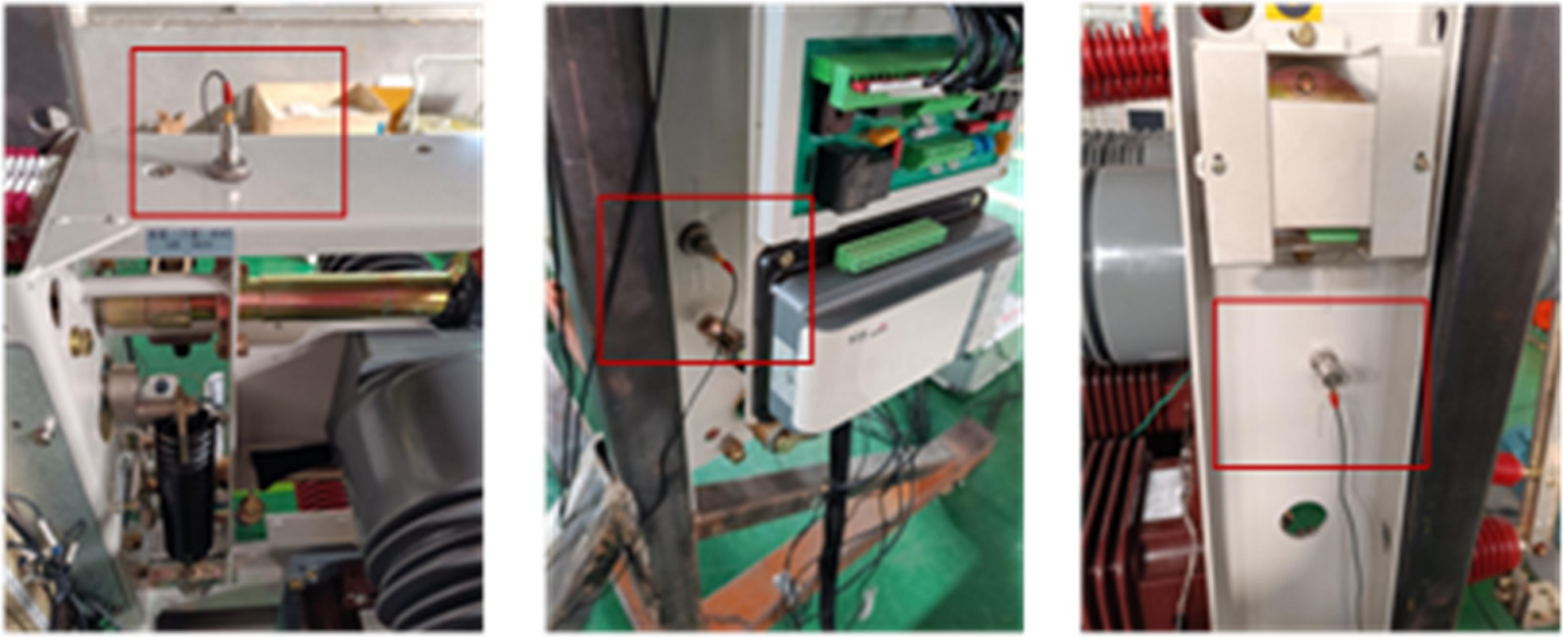
Installation position of vibration sensor.

To capture more comprehensive vibration signal information of the circuit breaker, three vibration sensors were positioned: one directly above the closing spring on the crossbeam (vibration sensor 1), another beneath the operating mechanism on the circuit breaker’s left side (vibration sensor 2), and the last on its right side (vibration sensor 3), as shown in Fig 10.

To diagnose faults in high-voltage circuit breakers accurately, it is necessary to first develop a standard fault database, and use this database to train various classifier models. During application, the trained model is used to classify and identify the collected data, and determine the type of circuit breaker fault. A typical fault database for basic circuit breakers has been established through preliminary research. This time, actual faults will be further simulated in a laboratory environment to build a more comprehensive fault database. The primary faults in high-voltage circuit breakers are mechanical in nature, with others being electrical or related to auxiliary circuits. This experiment simulated three types of mechanical failures, specifically: iron core jamming, spring fatigue, and component looseness.

### 4.2. Scene analysis

In feature extraction process, the hidden layer of the model is set to 5, and the input is 16 uncoded features. 440 sets of samples are selected for training, including data of normal operation state and fault states. In supervised fault classification experiments, there are four operating conditions, with labels B = [1,2,3,4] added respectively. 100 samples as the training set and 10 samples are selected as the test set for each operating condition. Considering the safety and feasibility of field application for power companies, this paper simulates different faults by changing the mechanical structure parameters of the original standard/well-functioning high-voltage circuit breaker. The specific fault types and data set number are shown in Table 3. 80% of the samples from the same type of fault date are selected randomly as the training set, while the rest is used for accuracy verification.

**Table 3:** Composition of high-voltage circuit breaker voiceprint detection dataset.

Circuit breaker states	Label	Number of training sets	Number of test sets
Normal	1	100	10
Spring fatigue	2	100	10
Iron core jamming	3	100	10
Component loosening	4	100	10
Total		400	40

The main types of faults in high-voltage circuit breakers are mechanical faults. This experiment simulated three types of mechanical faults, as shown in Table 4.

**Table 4:** Mechanical fault simulation plan.

Mechanical Fault Simulation Plan	Remarks
Iron core jamming	54g nuts are hung on the iron core of the opening and closing coils
Loose opening spring	The fixing nut of the opening spring is loose by 4mm
Loose components	Loosen the transmission bearing bolts

The training parameter settings of the deep learning method is shown in Table 5.

**Table 5:** Training parameter settings of deep belief networks.

Parameters	Value	Parameters	Value
Initial weight	Random	Pre training iterations	5
Initial bias	0	Number of hidden layers	5
Initial momentum	0.4	Number of input layer units	20
Steady-state momentum	0.9	Number of output layer units	20
Weight decay	0.0002	Reverse fine-tuning iterations	300
Learning rate	0.15		

In experimental calculations, the classification error rate of the deep belief network decreases with increasing layer number. However, when it reaches 6, the further added layer would only reduce the algorithm stability instead of the error rate. Therefore, layer number is set to 5 in this paper.

### 4.3. Feature extraction and fault diagnosis

In this calculation process, training error, testing error, and training time are used to measure the performance of algorithms and models. In the calculation of feature extraction, this article introduces the traditional deep belief network algorithm and a single deep automatic encoding algorithm for comparative analysis to verify the effectiveness of the proposed method [26]. Comparative results of feature extraction using different algorithms are shown in Table 4.

The calculation results in Table 6 indicated that the proposed method has smaller training and testing errors, and the difference in calculation time is not significant, meaning the accuracy of the calculation results had been significantly improved.

**Table 6:** Feature extraction results of different algorithms.

Algorithms	Time/s	Training error	Test error
The method of this paper	129.3	0.041	0.042
Traditional deep belief network algorithm	130.8	0.071	0.080
Single depth auto-encoding algorithm	132.5	0.082	0.086

### 4.4. Verification and analysis of actual examples

**Fig 11:**
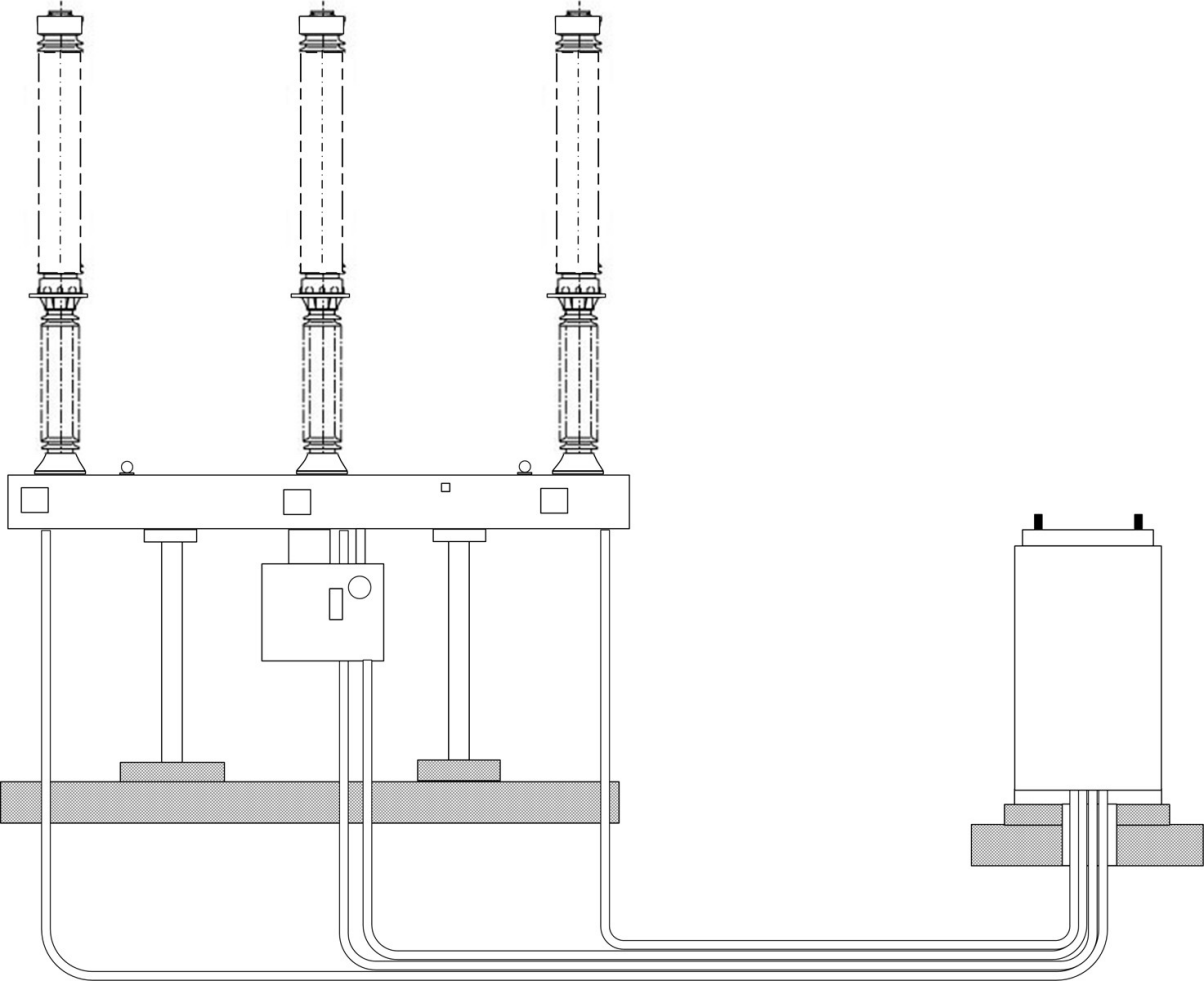
Structural diagram of high-voltage circuit breaker.

This article uses a YFGZ35 (EP) -40.5 circuit breaker from a 500kV substation in the southern Jiangsu region of China to obtain vibration information data of the high-voltage circuit breaker for development and analysis. The vibration information data collection device for the mobile circuit breaker developed by the institute was applied to obtain the historical operating data of the circuit breaker for the past three months as a test set. The data includes four different operating states of the circuit breaker, namely: normal state, spring fatigue, iron core jamming, and component loosening. The interval between data sampling time is 1ms. Based on the actual operation in substation, it was found that there is a significant frequency difference between the vibration voiceprint emitted by the circuit breaker itself and the background noise, making the latter one irrelevant to the outcome. The trained model in section 4.2 is applied to testing to verify the validity of the proposed method. In the test calculation, the output is the probability values of the samples of four different states. Considering calculation accuracy and time cost, the number of iterations is set to 250. The structural diagram of the high-voltage circuit breaker is shown in Fig 11.

**Fig 12:**
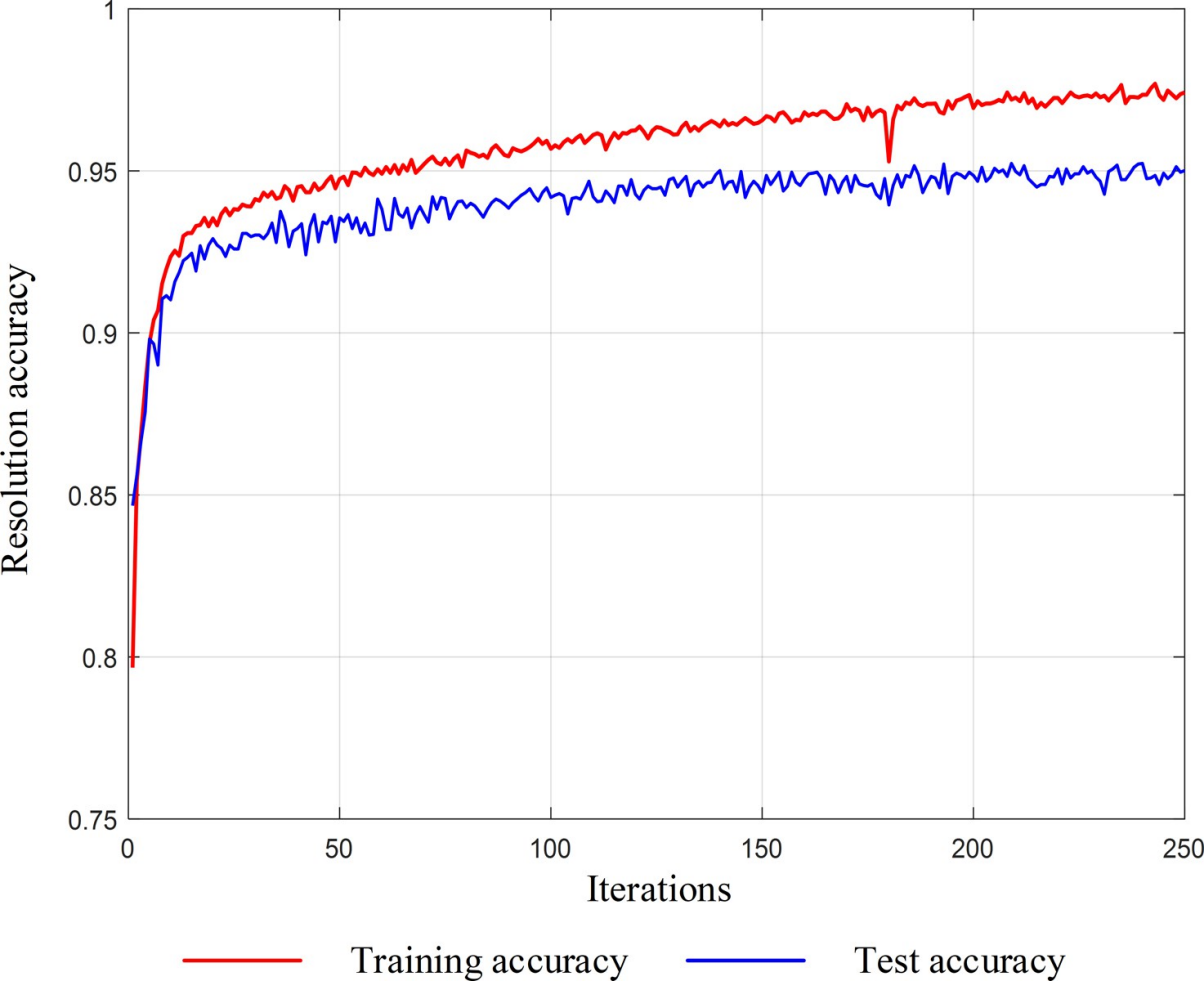
Overall accuracy of fault classification.

This paper inputs the test set into the trained model for calculation and analysis and uses average accuracy as the parameter to measure the accuracy of operating states diagnosis. The overall accuracy of the test set calculation is shown in Fig 12, which shows that the trained model is capable of verifying the operating states of the circuit breaker with the accuracy of surpassing 95%. Therefore, the validity of proposed method has been verified.

**Fig 13:**
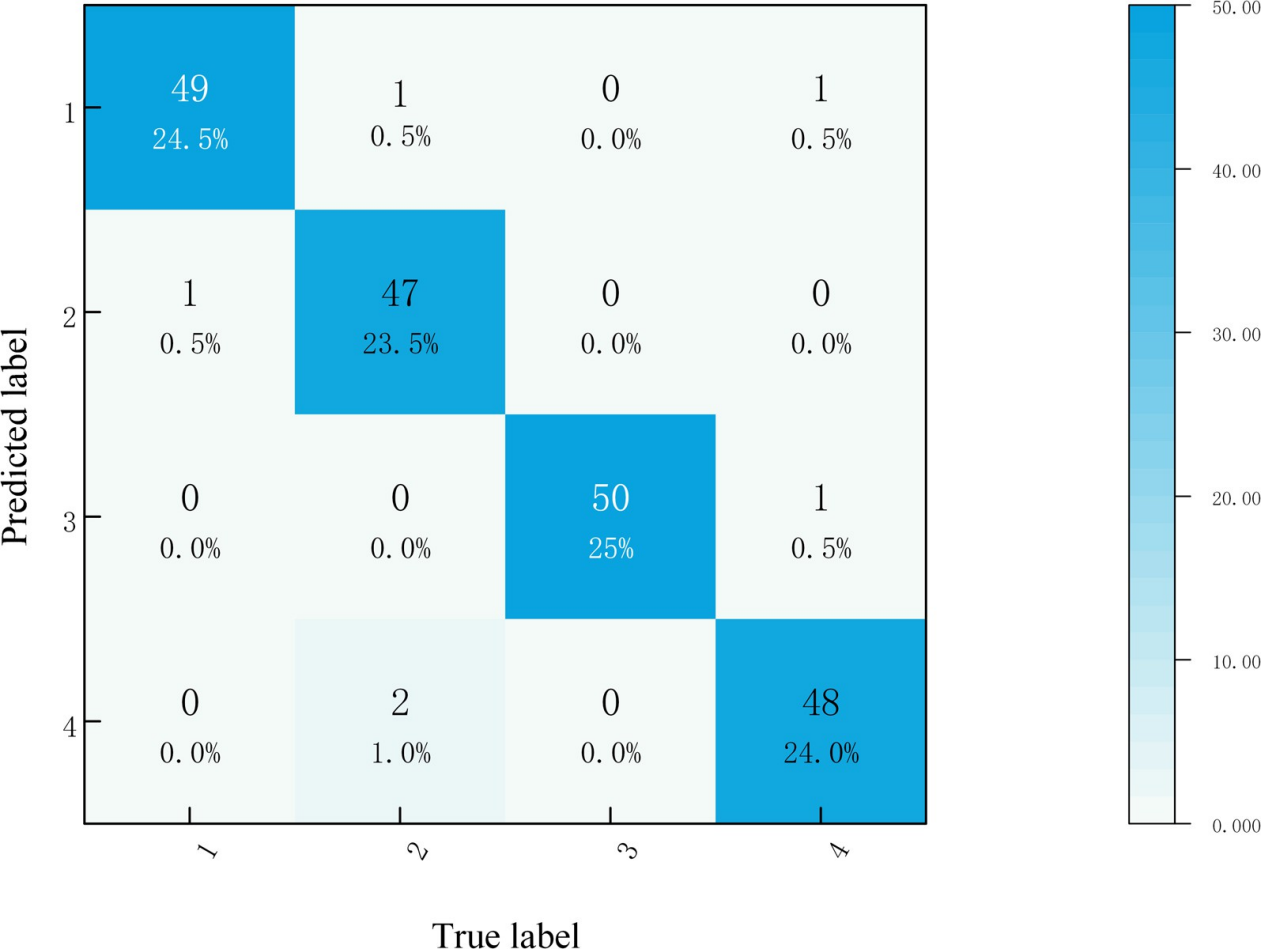
Fault classification confusion matrix.

Identification accuracy of different operation states is expressed by confusion matrix, as shown in Fig 13. The four types of operating states are normal state, spring fatigue, iron core jamming, and component loosening, which are represented by model 1, model 2, model 3, and model 4 respectively in the calculation. The identification accuracy of each four operating states is shown in Table 7.

**Table 7:** Identification accuracy of four operating states.

Model	Operating states	Identification accuracy
Model1	Normal	100%
Model2	Spring fatigue	95.1%
Model3	Iron core jamming	92.2%
Model4	Component loosening	93.1%
Average accuracy		95.1%

It could be seen from Table 7 that the average identification accuracy is 95.1%, which reaches 100% when identifying normal operating states in the given case study.

**Fig 14:**
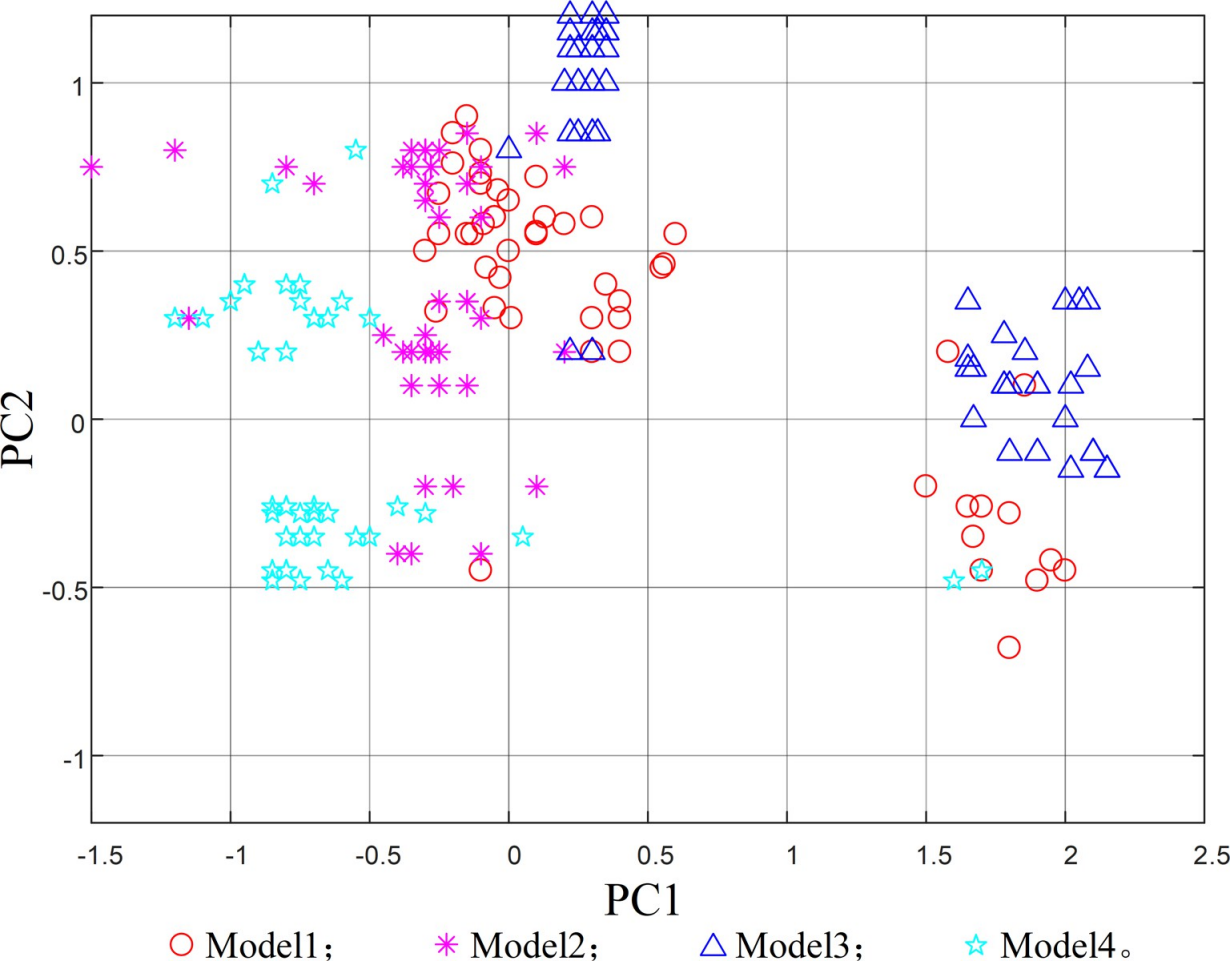
Scatter plot of raw feature mapping for test data.

**Fig 15:**
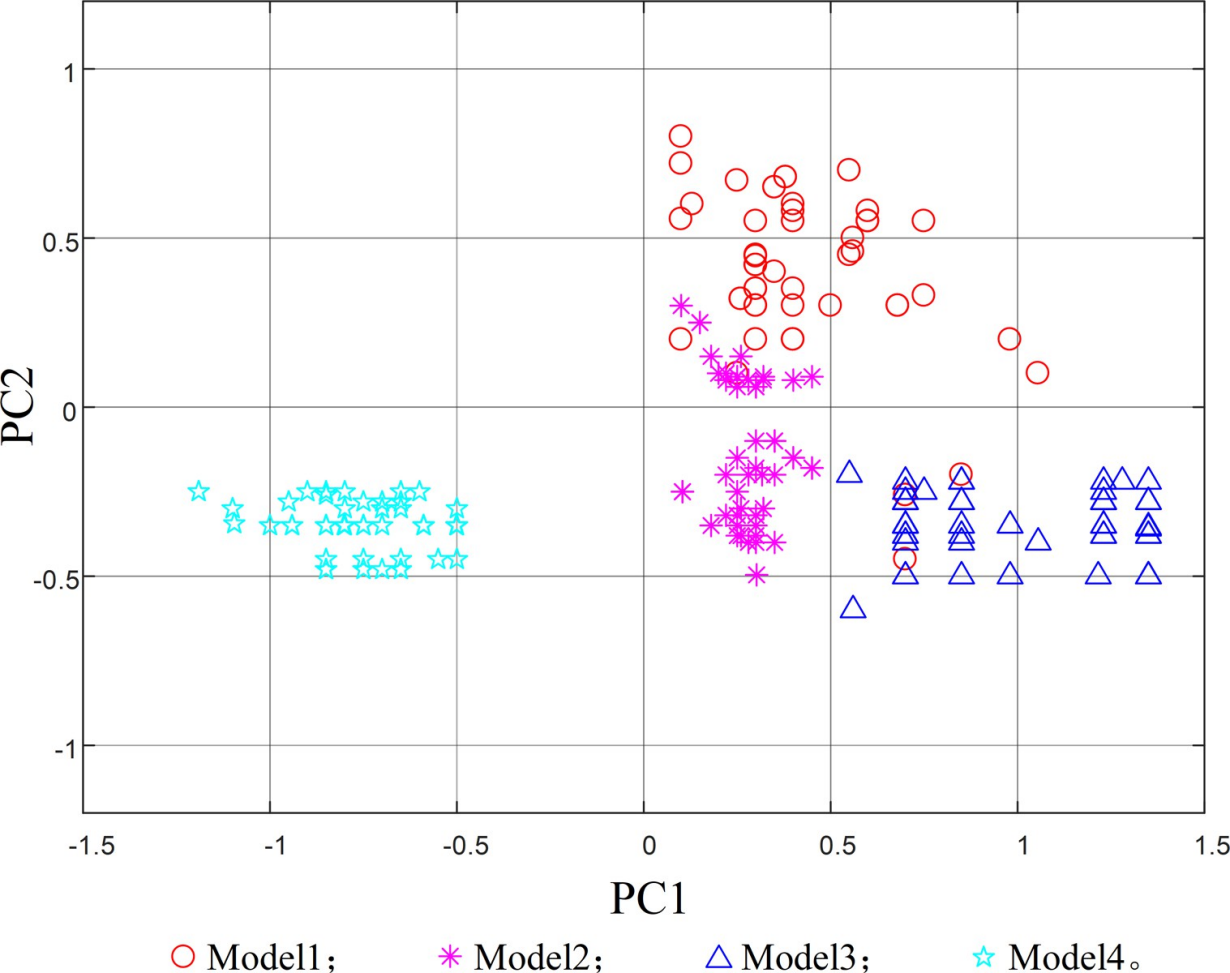
Top-level feature mapping scatter plot of test data.

When applying the model to extract features from the original data, the feature dimensions of each layer are high, making it difficult to observe. In order to observe features in a single graph, the principal component analysis mapping map is employed. In Fig 14, the scatter distribution map of the first two principal component analysis mapping maps of the original features of the training set data is shown. After feature extraction process, the visualization results of principal component analysis for different types of operating states are depicted in Fig 15.

From Fig 14, it can be seen that the data features of the four operating states are mixed together, making it difficult to perform effective feature extraction, yet compared with Fig 15, it is found that the feature extraction method proposed in this paper has a relatively obvious differentiation effect, meaning the four different operating states are basically separated, and effective operating state features can be extracted layer by layer from the original data, which further verifies the accuracy of the feature classification method proposed in this paper.

Among the many methods for identifying faults in high-voltage circuit breakers, the support vector machine method and pattern recognition method are widely used [27, 28]. In order to verify the advantages of the fault diagnosis algorithm proposed in this article, this section compares it with the support vector machine method and pattern recognition method to verify their accuracy in identifying three types of faults (iron core jamming, opening spring fatigue loosening, and component loosening). Comparative analysis mainly focuses on accuracy and calculation time, results are shown in Table 8.

**Table 8:** Calculation results of fault diagnosis using different methods.

Methods	Computing time/s	Average accuracy
Support vector machine method	231.6	84.6%
Pattern recognition method	274.5	89.3%
Proposed method in this paper	232.4	94.7%

The calculation results in Table 8 indicate that the support vector machine method takes less computation time, but the identification accuracy is too low. The pattern recognition method takes the longest computation time, and the identification accuracy is also not high. The method proposed in this article, although slightly longer in time than the support vector machine method, improves identification accuracy by about 10% and has significant advantages in computational accuracy. Therefore, the method proposed in this article has better accuracy and application value in fault identification.

The above calculation results indicates that applying the deep learning training model proposed in this paper to training can significantly improve the accuracy of circuit breaker fault monitoring. After unsupervised feature learning of circuit breaker data in section 4.3, the model parameters can adapt to the feature expression of the samples, improving the universality of high-level features. Therefore, in section 4.4, this method shows high accuracy and resolution when identifying the fault types of actual operating circuit breakers.

## 5. Conclusions

To solve the problem of insufficient operating status data samples of high-voltage circuit breakers, which poses difficulty in fault identification, this paper develops a high-voltage circuit breaker fault monitoring device and provides a mechanical fault monitoring method for high-voltage circuit breakers based on deep learning. The developed device facilitates the collection of vibration information data, simplifies the data processing process, and provides accurate fault diagnosis for circuit breakers. At the same time, the given data processing method preprocesses the original data of circuit breaker operation status, establishes the reconstruction model of deep confidence network through hidden layer reconstruction, and completes feature extraction; Then, using labeled samples to retrain the classification layer, a model for circuit breaker fault monitoring is obtained. The calculation of operational data obtained from substations shows that the device developed in this paper and the data processing method provided can accurately identify the fault status of circuit breakers, with an overall accuracy rate of surpassing 95%. It adeptly captures the diverse mechanical vibration patterns of high-voltage circuit breakers and proficiently executes fault diagnosis.

Compared with other algorithms, the algorithm proposed in this paper has an improved feature extraction accuracy and shorter computational time, which provides a better approach of mechanical failure monitoring of high-voltage circuit breakers.

On the basis of deep learning theory for fault diagnosis methods, locating faults with precision will be an important area of future research for the author.

## Supporting information

S1 Data (XLSX)
